# The safety and efficacy of second-generation cryoballoon ablation plus catheter ablation for persistent atrial fibrillation: A systematic review and meta-analysis

**DOI:** 10.1371/journal.pone.0206362

**Published:** 2018-10-25

**Authors:** Mengjiao Shao, Luxiang Shang, Jia Shi, Yang Zhao, Wenhui Zhang, Ling Zhang, Yaodong Li, Baopeng Tang, Xianhui Zhou

**Affiliations:** Department of Pacing and Electrophysiology, The First Affiliated Hospital of Xinjiang Medical University, Urumqi, Xinjiang, China; Albert Einstein College of Medicine, UNITED STATES

## Abstract

**Background:**

Growing evidence suggests that second-generation cryoballoon ablation (2G-CB) is effective in patients with persistent atrial fibrillation (PerAF). The cornerstone of atrial fibrillation (AF) ablation is pulmonary vein isolation (PVI). The purpose of this study was to summarize the available data on the safety and mid-term (≥ 12 months) effectiveness of a ‘PVI-only’ strategy *vs*. a ‘PVI-plus’ strategy using 2G-CB in patients with PerAF.

**Methods:**

We searched the PubMed, EMBASE and Cochrane library databases for studies on 2G-CB for PerAF. Group analysis was based on the ablation approach: ‘PVI-only’ versus ‘PVI-plus’, the latter of which involved PVI plus other substrate modifications. Studies showing clinical success rates at a follow-up (FU) of ≥ 12 months were included. Complication rates were also assessed. Data were analyzed by applying a fixed effects model.

**Results:**

A total of 879 patients from 5 studies were analyzed. After a mid-term FU of 27 months, the overall success rate of 2G-CB for PerAF was 66.1%. In the ‘PVI-plus’ group, the success rate was 73.8%. In the ‘PVI-only’ group, the success rate was 53.6%. No heterogeneity was noted among studies (*I*^*2*^ = 0.0%, *P* = 0.82). Complications occurred in 5.2% of patients (*P* = 0.93), and the rate of phrenic nerve (PN) injury was 2.8% (*P* = 0.14). Vascular assess complications were the most frequent at 1.6% (*P* = 0.33). No death or myocardial infarction was reported.

**Conclusion:**

‘PVI-plus’ involving 2G-CB seems to be safe and effective for treating PerAF.

## Introduction

Atrial fibrillation (AF) is the most common sustained cardiac arrhythmia in clinical practice [1]. AF is a well-known risk factor for thromboembolic events, silent cerebral infarcts, strokes, congestive heart failure and mortality in the general population and in high stroke risk patients, and it has become a major public health problem worldwide [2, 3]. Although the underlying mechanisms of AF are not yet fully understood, autonomic dysfunction, unbalanced inflammation/oxidative stress and renin-angiotensin system activation have all been shown to be related to AF [4–6]. However, the pulmonary vein (PV) is confirmed to be the most important and critical trigger for AF, and PV isolation (PVI) using catheter ablation is the cornerstone therapy for symptomatic AF refractory to antiarrhythmic drugs [7, 8]. The 2016 European Society of Cardiology (ESC) AF guidelines also recommend catheter ablation as a more effective therapy than antiarrhythmic drugs (AADs) for restoring and maintaining sinus rhythm (SR) in patients with symptomatic paroxysmal AF (PAF) and persistent AF (PerAF) [2].

Radiofrequency (RF) ablation is a well-established treatment for AF that achieves PVI by creating consecutive, transmural ‘point-to-point’ lesions with heat energy. Recently, cryoballoon (CB) ablation, which achieves PVI by a single-shot deployment of a CB with frozen energy, has become a substitute for RF ablation, as CB ablation has the advantage of being an easier and faster ablation procedure than RF ablation [9–11]. Several previous studies have shown that the efficacy and safety of CB ablation therapy are comparable to those of RF ablation in patients with PAF [10–13]. Compared with first-generation equipment, second-generation CB (2G-CB) ablation devices have preponderant cooling capacity and seem to reduce the procedure duration [14–16]. However, 2G-CB ablation to achieve PVI has some shortcomings, including an inability to perform ablation of roof linear (RL) lesions, complex fractionated atrial electrograms (CFAEs) and non-PV triggers [17]. On the other hand, early studies of PVI involving only patients with PerAF revealed suboptimal success rates [18]. Therefore, the ‘PVI-plus’ ablation strategy that combines 2G-CB ablation to achieve PVI and RF ablation and addresses additional cardiac substrate modification and extra-PV lesions during the same surgery might be a better strategy for treating PerAF and long-standing PerAF [2, 19]. However, the effectiveness and safety of the ‘PVI-plus’ ablation strategy have not been sufficiently discussed.

To this end, we executed a pooled analysis and meta-analysis of data from existing studies and trials investigating the efficacy and safety of ‘PVI-plus’ ablation vs. ‘PVI-only’ ablation in patients with PerAF.

## Methods

Our systematic literature search was performed according to the Meta-Analysis of Observational Studies in Epidemiology (MOOSE) guidelines [20] and conducted using a predetermined protocol by the Preferred Reporting Items for Systematic Reviews and Meta-Analyses (PRISMA) statement [21].

### Literature search strategy

The search strategy was conducted in the PubMed, EMBASE and Cochrane library databases until September 1, 2018. The search terms were as follows: (“Catheter Ablation” OR “Cryosurgery” OR “Second-Generation Cryoballoon”) AND (“Atrial Fibrillation” OR “Persistent Atrial Fibrillation”). No restrictions were applied on regions or languages. We also manually searched the reference lists of all publications and review articles to identify other relevant studies.

### Study selection

Two investigators (JS and LXS) independently scanned all the titles and abstracts to identify studies that met the inclusion criteria and extracted data from these studies. Discrepancies between reviewers were resolved by discussion with a third reviewer (MJS).

The inclusion criteria were as follows: (1) involved patients with drug-refractory symptomatic PerAF who underwent 2G-CB; (2) involved patients who were treated with a ‘PVI-only’ strategy or a ‘PVI-plus’ strategy using 2G-CB (during surgery, PVI and PVI-plus had to be carried out simultaneously); (3) reported 1-year clinical success rates; (4) involved follow-up (FU) periods longer than 12 months; (5) published as a full-text article; and (6) provided data regarding efficacy and safety.

Studies were excluded for the following reasons: (1) conference abstracts, case reports, case series, editorials, or review articles. (2) They did not report clinical success rates. (3) The maximum FU period was shorter than 12 months. (4) They were animal or in vitro studies.

### Data extraction and quality assessment

Two primary investigators (JS and LXS) independently evaluated and extracted data from each study. Data on the first author, publication year, study population, age, sex ratio, CHA2DS2-VASc score, underlying disease, medication usage and ablation strategy were collected using a predesigned electronic form. All studies were reviewed twice, and disagreements were discussed and resolved by consensus in a meeting with a third investigator (MJS). We defined the primary outcome criterion as no episode of AF or any atrial arrhythmia lasting longer than 30 s without administration of AADs after a single ablation procedure using 2G-CB with an FU period of at least 12 months (considering an initial blanking period (BP) of 3 months). Group analysis was performed based on the ablation strategy: ‘PVI-only’ strategy versus ‘PVI-plus’ strategy, the latter of which involved PVI plus another substrate modification. The secondary outcomes were complication rates, including the phrenic nerve (PN) palsy (PNP) rate, the rates of cardiac and vascular assess site complications (including hematomas, pseudoaneurysms, and fistulas), and the rates of other complications (pericardial effusions and/ or tamponade, PV stenosis or atrioesophageal fistula).

Whenever the data of interest were not available in the literature, the investigators tried to contact the authors by email to obtain the data. We described incomplete data as “not reported” in our manuscript.

We used the Newcastle-Ottawa Scale (NOS) to further evaluate the quality of the observational studies, and a NOS score ≥ 7 was considered good quality [22].

### Statistical analysis

Statistical analysis was performed using the Cochrane RevMan version 5.3 software (The Cochrane Collaboration, UK). The results are reported as weighted mean differences and relative risks (RRs) with 95% confidence intervals (CIs) for continuous and dichotomous outcomes, respectively. Heterogeneity was assessed using the *I*^*2*^ test, Cochran’s Q statistic and the *I*^*2*^ index. *I*^*2*^ values of 25%, 25–50%, or 50% indicated low, moderate, or high heterogeneity, respectively [23]. Funnel plot analysis was used to evaluate potential publication bias. In all analyses, a *P* value < 0.05 was considered statistically significant.

## Results

### Study characteristics

**Fig 1:**
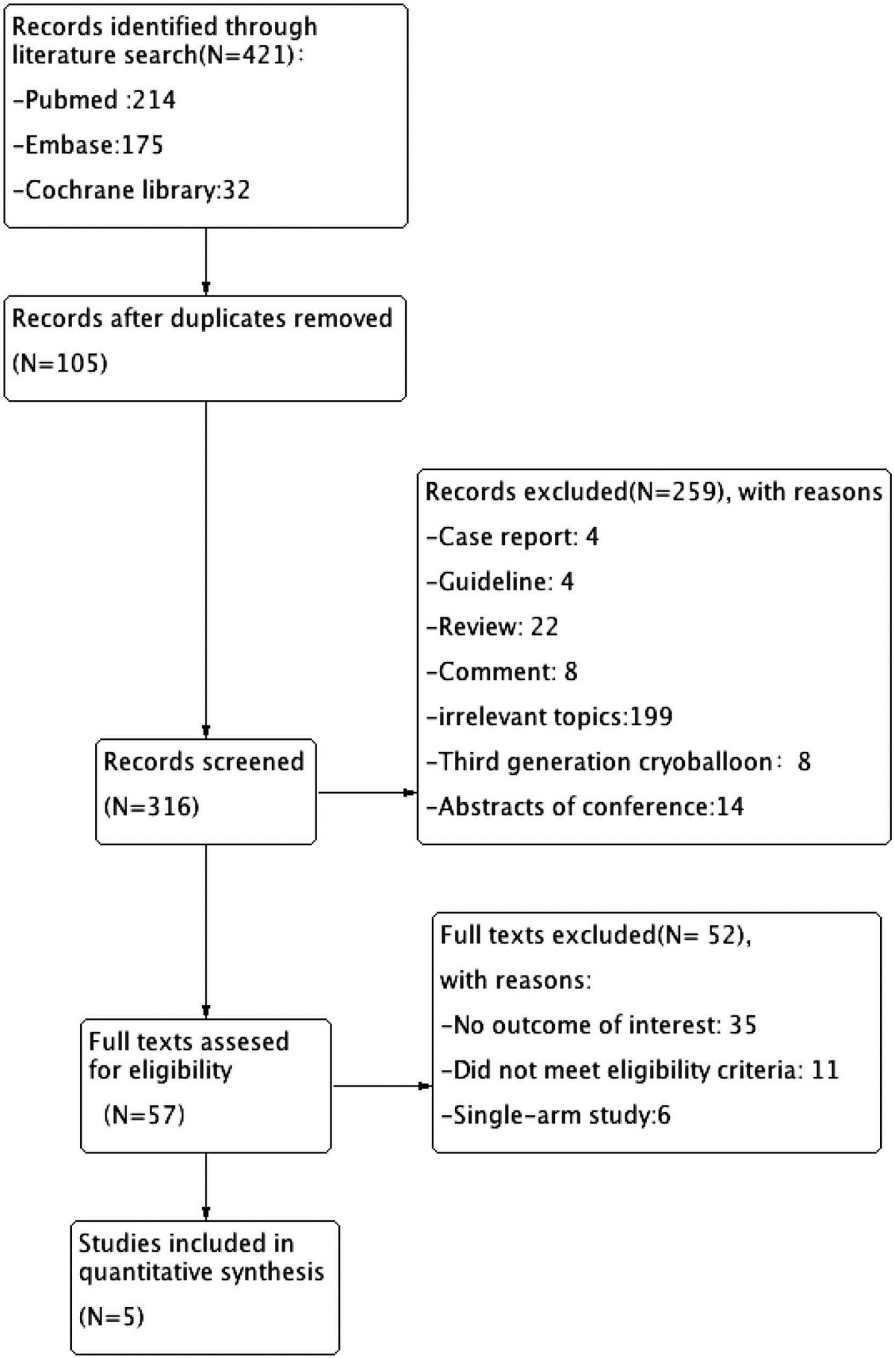
Flow diagram of the stages of the literature search.

Finally, as illustrated in **Fig 1**, four studies involving a total of 879 patients who underwent ablation with 2G-CB for PerAF were enrolled in this study. The safety and efficacy of a combination of extra-PV lesions (linear ablations and/or atrial substrate modifications) or PVI alone with 2G-CB for PerAF ablation were identified.

The characteristics of the included studies are described in Table 1. All of the included patients completed at least one year of FU. Five studies were analyzed [24–28]. Unfortunately, we did not find randomized controlled trials meeting the inclusion criteria. The quality and bias of the included studies are shown in Table 2. Four full-text studies had an NOS score of 8, and the other study had an NOS score of 7.

**Table 1:** Main characteristics of the included studies.

Study	Akkaya et al.^[^^24^^]^2018	Akkaya et al.^[^^25^^]^2017	Su et al.^[^^26^^]^2016	Aryana et al.^[^^27^^]^2015	Aryana et al.^[^^28^^]^2018	Overall
**Location**	Germany	Germany	USA China	USA Canada Brazil	USA Brazil	-
**Design**	Prospective	Retrospective	Retrospective	Retrospective	Prospective	-
**Total**	101	111	225	1196	390	2023
**FU duration (months)**	37 (31/42)	27 (15/37)	12	12	12	20
**AF type**	PAF/PerAF	PerAF	PAF/PerAF	PAF/PerAF	PerAF	-
**n (PerAF)**	61	111	137	180	390	879
**Age (years)**	64 (55/70)	62 (54/69)	Not reported	63 ± 10	63 ± 10	63
**Male (%)**	66	77	Not reported	70	65	69.50%
**CHA2DS2-VASc-Score**	1.9 ± 1.4	1.8±0.8	Not reported	2.1 ± 1.5	2.6 ± 1.5	2.1
**BMI, kg/m**^**2**^	28.4 (25.5/31.8)	28.3 (26.0/32.3)	Not reported	Not reported	32± 7	29.6
**Hypertension, n (%)**	78 (77.2)	81 (73.0)	Not reported	115 (64)	269 (69.0)	70.80%
**Diabetes, n (%)**	4 (13.9)	17 (15.3)	Not reported	22 (12)	83 (21.3)	15.63%
**Structural heart disease, n (%)**	10 (9.9)	10 (9.0)	Not reported	24 (13)	84 (21.5)	13.35%
**LVEF (%)**	62 (57/62)	62 (57/62)	Not reported	57 ± 10	54.5±12.5	58.88
**LA (mm)/ LA area (cm2)**	22.1 (19.7/25.6)	44 (41–48)	Not reported	44 ± 9	45.5±8	44.5
**OAC, n (%)**	101 (100)	Not reported	Not reported	133 (74)	346 (88.7)	87.57%
**Vitamin K antagonists**	46 (45.5)	Not reported	Not reported	222 (29)	Not reported	37.25%
**Novel oral anticoagulants**	55 (54.5)	Not reported	Not reported	351(46)	Not reported	50.25%
**Antiarrhythmic therapy, n (%)**	70 69.3)	75 (67.5)	31(22)	429 (56)	117 (30)	48.96%
**Amiodarone**	14 (13.9)	21 (18.9)	Not reported	160 (21)	Not reported	17.93%
**Sotalol**	1 (1.0)	2 (1.8)	Not reported	55 (7)	Not reported	3.27%
**Class I**	38 (37.6)	31 (27.9)	Not reported	134 (18)	Not reported	27.83%
**Dronedarone**	17 (16.8)	21 (18.9)	Not reported	130 (17)	Not reported	17.57%
**Other**	Not report	Not reported	Not reported	20 (3)	Not reported	3.00%
**Monitor type during FU**	7-d Holter ECG, FU visits at 3 and 6 M	7-d Holter ECG recordings	Cardiac monitors at 3, 6, and 12 M	24-h Holter ECG at 6 W and 3, 6, and 13 M	2 W Mobile cardiac telemetry monitoring was performed at 6 W, 3~6 M, 13 M, and 18 M	-

All data were obtained from the overall study population.

PAF: paroxysmal atrial fibrillation, PerAF: persistent atrial fibrillation, PVI: pulmonary vein isolation, 2G‐CB: second-generation cryoballoon, FU: follow-up, OAC: oral anticoagulant, LVEF: left ventricular ejection fraction, LA: left atrium diameter, ECG: echocardiography, NA: not available, D: days, M: months, W: weeks.

Novel oral anticoagulants, including dabigatran, rivaroxaban and apixaban.

Other antiarrhythmic therapies, including dofetilide.

**Table 2:** Quality and bias of the included trials.

Source	Akkaya et al.^[^^24^^]^	Akkaya et al.^[^^25^^]^	Su et al.^[^^26^^]^	Aryana et al.^[^^27^^]^	Aryana et al.^[^^28^^]^
**Year of publication**	2018	2017	2016	2015	2018
**Selection bias**					
Representativeness of the exposed cohort	★	★	★	★	★
Selection of the nonexposed cohort	★	★	★	★	★
Ascertainment of exposure	★	★		★	★
Demonstration that the outcome of interest was not present at the start of the study	★	★	★	★	★
**Comparability**					
Comparability of cohorts on the basis of the design or analysis	★	★	★	★	★
**Outcomes**					
Assessment of outcome	★	★	★	★	★
Follow-up of sufficient duration for outcomes to occur	★	★	★	★	★
Adequacy of the follow-up of cohorts	★	★	★	★	★

The patients were predominantly male (69.5%); 70.1% had hypertension, 15.6% had diabetes, and 13.35% had structural heart disease. In all the studies, 87.6% of patients were treated with oral anticoagulants for PerAF. The mean CHA2DS2-VASc score was 2.1.

The mid-term FU (≥ 12 months, considering an initial BP of 3 months) occurred at 27 months. Regarding monitoring during FU, 2 studies implemented 24-h Holter recordings during FU, while two studies recorded data from 7-day Holter or external cardiac event recorders; the other studies recorded data from 2-week mobile cardiac telemetry monitoring performed at 6 weeks, 3~6 months, 13 months, and 18 months.

### Publication bias

**Fig 2:**
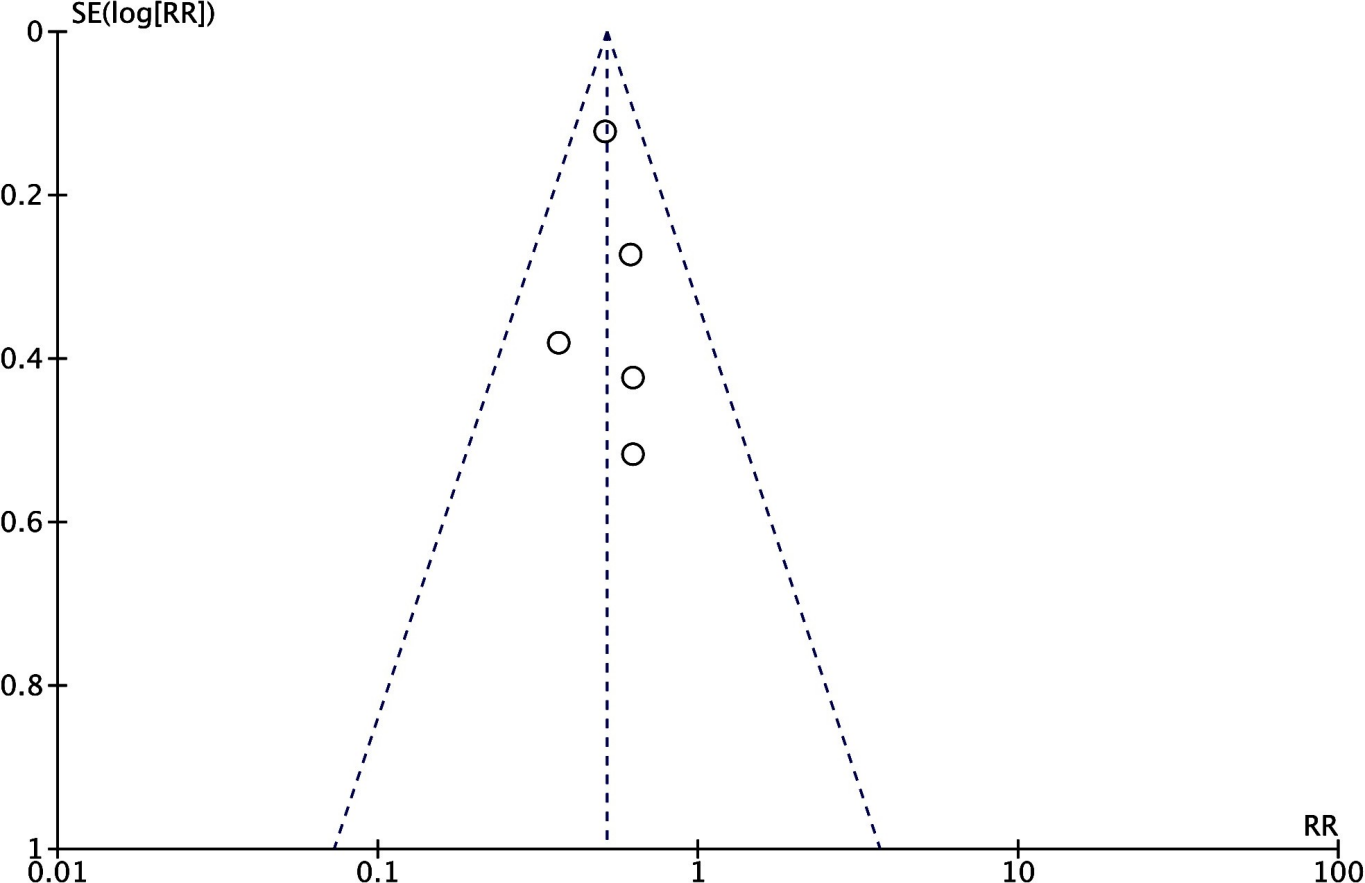
Funnel plot for the assessment of publication bias in the primary outcome. Effect size is plotted on the x-axis, and the SE is on the y-axis. RR: relative risk; SE: standard error.

Regarding the proportion of patients who were free from AF recurrence during FU, all the included studies had publication bias that was approximately symmetrical on visual inspection of the funnel plot **(Fig 2).**

### Ablation strategy

In 3 studies, a bonus freeze protocol was implemented (Table 3) with an additional freezing cycle applied to each PV after successful PVI. Subgroup analysis to compare freezing protocols was not performed because the majority of patients were treated with a bonus freeze protocol. All studies (including 543 patients) performed PVI plus other linear ablations or substrate modifications. There was no significant methodological heterogeneity in terms of patient characteristics and ablation strategies (*I*^*2*^ = 0%, *P* = 0.82).

**Table 3:** Ablation strategy and freezing protocol.

Study	Ablation strategy	Freezing protocol	Procedure times(minutes)	Fluoroscopy time(minutes)	Intra ECV (n)
Akkaya et al.^[^^24^^]^2017	PVI+ roof line + CIA	Each PV freezing cycle lasted 180 s + a bonus freeze of 150~180 s	120 (102 of 147)	20 (16 of 27)	36
Akkaya et al.^[^^25^^]^2017	PVI + roof line+ CIA	Each PV freezing cycle lasted 180 s + a bonus freeze of 240 s	102 (79/120)	16 (12/24)	40
Su et al.^[^^26^^]^2016	PVI + roof line+ substrate modification	Each PV freezing cycle lasted 180 s + a bonus freeze of 180 s	13200B136	4.2± 2.2	Not reported
Aryana et al.^[^^27^^]^2015	PVI + non-PV triggers	1~3 Freezes to each PV, each between 120 and 360 s	145 ± 49	29 ± 13	Not reported
Aryana et al.^[^^28^^]^2018	PVI+PWI	1~2 Freezes to each PV, each between 120~180 s.	188 ± 42	28 ± 9	178

PVs: pulmonary veins, s: second, CIA: cavotricuspid isthmus ablation, CFAEs: complex fractionated atrial electrograms, non-PV triggers: linear and/or atrial substrate ablation, Intra ECV: intraprocedural electrical cardioversion, PWI: posterior left atrial wall.

The result of the study by Aryana et al. [27] demonstrated the ablation of non-PV triggers in 12.8% of all patients treated with 2G-CB without further specifications. The mean procedure time was 131.9 ± 7.2 min in the ‘PVI-plus’ group and 124.2 ± 13.2 min in the ‘PVI-only’ group. No interaction was found between the ablation approach and clinical success rate (*P* = 0.47). However, the results of the study by Su et al. [26] confirmed that application of 2G-CB could achieve large-area atrial substrate modification mostly with left atrial (LA) roofline and Coumadin-ridge ablation (atrial tissue between the LA appendage and left superior PV). Akkaya et al. [25] showed that among patients with PerAF and LA enlargement, PVI with 2G-CB in addition to the creation of RLs may be a viable alternative to point-by-point RF ablation, as indicated by a lower arrhythmia recurrence rate with this strategy than with the ‘PVI-only’ strategy. Aryana et al. [28] demonstrated that ablation of PVI in conjunction with isolation of the posterior LA wall is associated with improved clinical outcomes in certain patients with AF.

### Primary outcome

#### Recurrence

**Fig 3:**
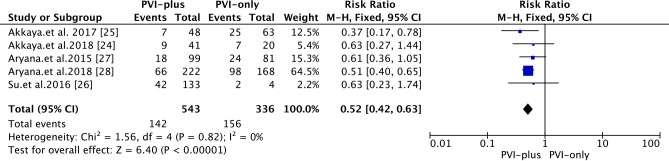
Forest plot of the primary efficacy outcome of 2G-CB for PerAF at a mid-term FU.

Data from 5 studies were included. At a mid-term FU of 27 months, the overall success rate of 2G-CB in patients with PerAF was 66.1% **(Fig 3)**. In the ‘PVI-plus’ group, the success rate of 2G-CB in patients with PerAF was 73.8%. In the ‘PVI-only’ group, the success rate of 2G-CB in patients with PerAF was 53.6% [RR: 0.52; 95% CI 0.42~0.63, *P*<0.00001].

The heterogeneity among studies was not significant (*I*^*2*^  =  0%, *P* = 0.82). The rate of AAD use at the FU was 48.96%. The rate of AAD use at the FU was reported in 4 studies.

However, because of the lack of correlated studies, we could not analyze subgroups in detail. Meanwhile, the heterogeneity among studies was not significant. Categories including PerAF (short-term PerAF, long-standing PerAF or unspecified PerAF), FU strategy (24-h Holter monitoring vs. 7-day Holter monitoring or event recording), and AF population (PerAF alone, mixed AF, such as PAF and PerAF) could not be included in further subgroup analyses.

#### One-year recurrence

**Fig 4:**
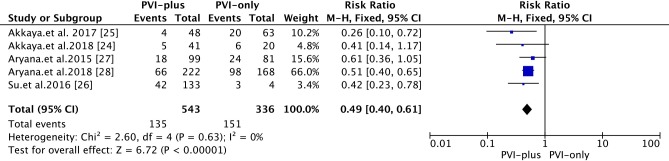
Forest plot of the primary efficacy outcome of 2G-CB for PerAF at the one-year FU.

At the one-year FU, compared with that in the ‘PVI-plus’ group, the one-year success rate associated with 2G-CB in the ‘PVI-only’ group was 55.1% (vs. 75.1% in the ‘PVI-plus’ group) [RR 0.49; 95% CI 0.40~0.61, *P*<0.00001] **(Fig 4)**. The heterogeneity among studies was not significant *(I*^*2*^* * =  0%, *P* = 0.61).

### Secondary outcomes

#### Overall complications

**Fig 5:**
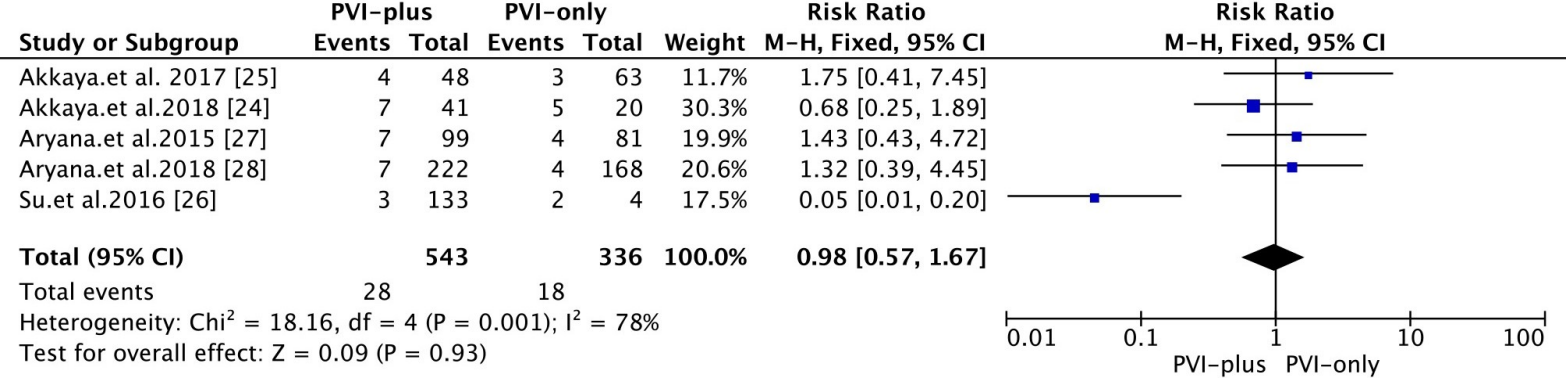
Forest plot of the overall complications from 2G-CB in patients with PerAF.

The overall complication rate was 5.2% **(Fig 5)**. Phrenic nerve palsy (PNP)/PN injury were the most frequent complications. Compared with that in the ‘PVI-plus’ group, the complication rate associated with 2G-CB in the ‘PVI-only’ group was 5.4% (*vs*. 5.2% in the ‘PVI-plus’ group) [RR 0.98; 95% CI 0.57~1.67, *P* = 0.93].

#### Phrenic nerve palsy

**Fig 6:**
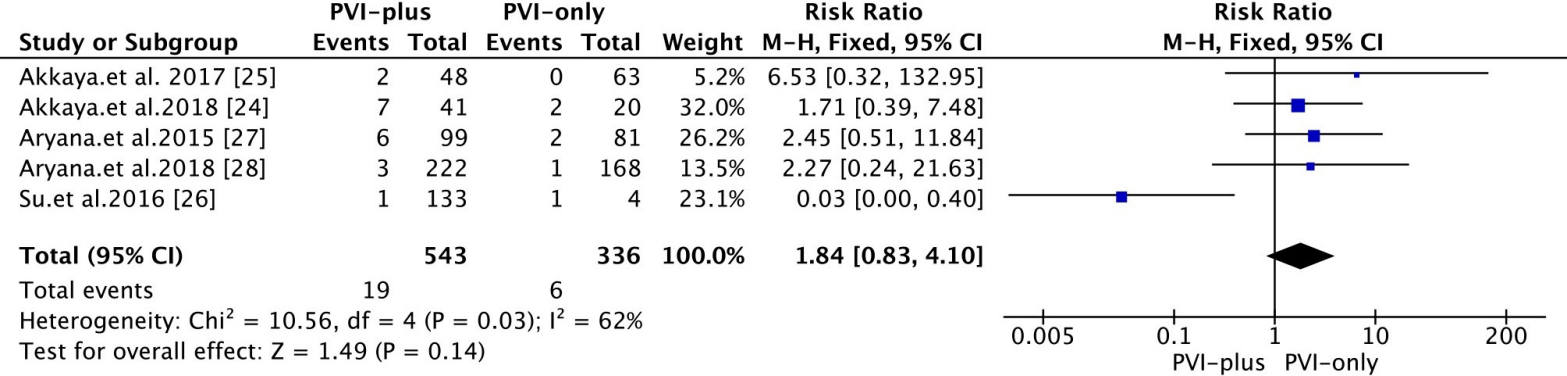
Forest plot of PNPs after 2G-CB in patients with PerAF.

PNP was found in all studies. Transient PNP was defined as PNP that resolved by discharge. Persistent PNP was defined as PNP that persisted beyond discharge. The overall rate of PNP was 2.8% **(Fig 6)**. Compared with that in the ‘PVI-plus’ group, the rate of PNP associated with 2G-CB in the ‘PVI-only’ group was 1.8% (*vs*. 3.5% in the ‘PVI-plus’ group) [RR 1.84; 95% CI 0.83~4.10, *P* = 0.14]. Two studies reported persistent PNP [27,28] that recovered during FU (the maximal time to PN recovery was not reported).

#### Vascular access site complications

**Fig 7:**
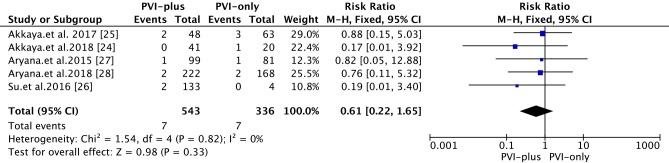
Forest plot of vascular assess site complications from 2G-CB in patients with PerAF.

The overall rate of vascular access site complications was 1.6% **(Fig 7)**. Compared with that in the ‘PVI-plus’ group, the PNP complication rate associated with 2G-CB in the ‘PVI-only’ group was 2.1% (*vs*. 1.3% in the ‘PVI-plus’ group) [RR 0.61; 95% CI 0.22~1.65, *P* = 0.33]. In one study, 5 patients had a groin complication with a hematoma that required more than 2 weeks for full recovery, and 3 patients had a pseudoaneurysm that required a thrombin injection for patient recovery [26]. Another study reported that 3 patients had minor groin hematomas [24].

#### Other complications

No death, myocardial infarction, PV stenosis or atrioesophageal fistula related to the procedure was reported. One patient had a perforation that resulted in cardiac tamponade requiring pericardiocentesis and immediate surgical treatment [26]. One patient had a stroke after ablation and was treated conservatively [25].

## Discussion

### Principal findings

In this detailed meta-analysis and systematic review of trials, we evaluated different approaches to 2G-CB in patients with PerAF. The main findings are as follows: (i) the ‘PVI-plus’ strategy had comparable clinical efficacy and safety as the ‘PVI-only’ strategy; (ii) we detected a reduction in the proportion of recurrent AF when either RL ablation, ablation of CFAEs or substrate modification was executed in addition to ablation with PVI; and (iii) compared with ‘PVI-plus’ involving 2G-CB, ‘PVI-only’ involving 2G-CB had similar rates of severe complications.

### Efficacy

We used different approaches for 2G-CB. In the ‘PVI-plus’ group, the success rate of 2G-CB in patients with PerAF was superior to that in the ‘only PVI’ group. Additional ablation can isolate other targets and reduce recurrence. Previous studies have shown that the expression of circulating miRNAs changes after ablation of AF, and these miRNAs participate in atrial electrical remodeling and fibrosis [29–30], which might be one of the reasons why additional matrix ablation reduces the recurrence of PerAF. However, such ablation may be arrhythmogenic [5].

The STAR AF II trial [31] reported no added advantage from a more extensive ablation than PVI with RF for PerAF. This study showed a success rate of 68.9% at a mean FU of 16.7 months. Compared with the results of recent studies involving cohorts treated with RF energy, 2G-CB may be the optimal treatment strategy. In our study, the mid-term FU was 27 months, and there was a greater than 70% success rate at the mid-term FU. However, in other studies evaluating PVI alone for PerAF, such as a previous meta-analysis, a success rate of only 51.9% was observed [32]. The improved success rates noted in our meta-analysis are likely multifactorial and related to differences in the patient population and the use of wide RL ablation, which incorporates some substrate and technological changes.

### Ablation strategy

Patients with PerAF were recognized to have more advanced atrial remodeling than those with paroxysmal AF [33]. Until now, the main strategy for ablation of PerAF was atrial substrate modification (in addition to PVI) to achieve acceptable success rates. Importantly, similar to the results of our study, some meta-analyses have demonstrated improved ablation outcomes when substrate ablation (CFAE or linear ablation) was performed in addition to PVI in PerAF patients [34, 35].

Linear ablations (the most common type of ablation) were successful in this study when cryoenergy freezes lasting 180 s were applied along the LA roof by caudocranial ascending activation and a conduction delay next to the ablation line of > 120 s when pacing at the right atrial upper septum during SR [25]. In another study involving AF without conversion to SR after PVI, additional LA rooflines were created with point-by-point lesions using contact force (CF) catheters. The acute endpoint was the elimination of local bipolar electrograms, with 20- to 40-s lesions or with a force-time integral > 400 s. CF ablation was performed for documented atrial flutter prior to or during the procedure [24]. This study reported that when AF ablation required more than CB PVI, an ablation catheter and technique were used on additional lesions. Catheter maneuvers necessary to achieve electrical blockage, substrate modifications and clinical outcomes were recorded. The 11 methods of CB catheter substrate modification that are described in this study included all the extra-PV lesion sets applied for 120 s~180 s at each location [26]. In the other study, LA linear ablations and substrate modifications, including cavotricuspid isthmus ablation and substrate ablation of CFAEs and other non-PV triggers, were performed. RF ablation was performed using an externally irrigated, non-force-sensing catheter with a 3.5-mm tip. Power was limited to 40 W over the anterior walls and 30 W over the posterior walls [27]. At the end of the study, using a high-density mapping catheter and the available data, between one and two 120~180 s cryoapplications were delivered to each PV with guidance by time to PVI. Once PVI was achieved, the cryoballoon was used to perform PWI in patients undergoing this treatment. PVI+PWI can be achieved safely and effectively using the cryoballoon. However, the latter required adjunct RF ablation for completion of PWI in approximately one-third of the patients [28].

Therefore, we evaluated PVI, which is the cornerstone treatment for ablation of AF, and atrial substrate modification. This combination had a higher success rate than other methods. We found that ‘PVI-plus’ with 2G-CB for the treatment of PerAF was an effective ablation strategy that potentially reduced the procedure and fluoroscopy times. The improved effect with 2G-CB is likely due to structural improvements in the device, which optimized the refrigerant flow and distribution, providing a larger, more uniform freezing zone at the entire distal hemisphere of the balloon irrespective of balloon orientation and enabling a shorter application time [36, 37].

### Safety

In terms of overall outcomes, no deaths, myocardial infarctions or clinical cerebral emboli were reported in a total of 879 patients. The overall complication rate was 5.2% and mainly included PNPs (2.8%) and vascular access site complications (1.6%).

Previous studies have reported similarly high rates of PNP (up to 5.4%) and vascular complication [38]. Casado-Arroyo et al. [39] reported that 2G-CB was more likely to cause PNP than other methods, possibly due to the larger cooling surface area, ablation area being more adjacent to the PN, and deeper damage foci. Moreover, the percentage of complications excluding PNP seemed to be lower with 2G-CB than with RF ablation [40]. On the other hand, the incidence of pericardial effusion and/or tamponade was very low with 2G-CB and was consistently lower than the previously reported incidence with RF [41].

### Heterogeneity analysis

In this study, significant heterogeneity was observed in the incidence of total complications and the rate of PNP. Due to the lack of correlated studies, we could not analyze detailed subgroups. On the other hand, the enrolled populations in the included studies may have been affected by the selection bias associated with a single-center experience and the preferences of different centers that refer patients for AF ablation because different centers used different protocols and tools, which ultimately resulted in substantial heterogeneity.

In addition, there was no significant heterogeneity in recurrence and one-year recurrence in the overall population, and funnel plot analysis did not provide evidence of significant publication bias. Therefore, it was believed that the included studies had sufficient similarities. In conclusion, the outcome of the meta-analysis was reliable.

### Previous meta-analysis

A previous and similar meta-analysis [9] summarized data on the safety and mid-term efficacy of PVI using 2G-CB in patients with PerAF. A total of 11 studies were analyzed. After FU, 68.9% of patients were free from recurrence. Complications occurred in 5.5% of patients.

Compared with the abovementioned meta-analysis, our meta-analysis used different inclusion criteria and required ‘PVI-only’ and ‘PVI-plus’ to be performed simultaneously. However, studies that included patients treated with other substrate modifications were permitted as long as PVI was performed using 2G-CB at least once during surgery. Similarly, we used the inclusion criterion that PVI was performed using 2G-CB. Thus, only 4 studies were included in this review.

### Limitations

The limitations of this article include the following:

1. We did not find randomized trials that were eligible for this analysis, and the total sample size was not sufficient.
2. Heterogeneity among studies was significant in total complications and the rate of PNP, and we could not perform a more detailed subgroup analysis. In addition, the inclusion of only published studies may have resulted in publication bias toward more favorable ablation outcomes from more experienced centers with variable FU and assessment of arrhythmia recurrence. For example, in 2/4 studies, postablation monitoring was performed with only 24-hour monitors, and previous studies have shown that continuous monitoring and telemonitoring to detect clinical and subclinical AF events are more effective than 24-hour monitoring [42]. Moreover, the use of AADs varied between studies, and postablation monitoring was performed with only 24-hour monitors.

1. Two studies included in this meta-analysis may have involved the same study population. We tried to contact the authors via email to evaluate the data. Unfortunately, we did not receive any response from those authors.
2. The studies we included were not direct studies of PVI-only *vs*. PVI-plus adjunctive ablation strategies for PerAF; rather, we extracted the data that we were interested in. In addition, some of the data needed to be calculated based on the results of the studies.

## Conclusions

In conclusion, this meta-analysis suggests that ‘PVI-plus’ involving 2G-CB for the treatment of patients with PerAF seemed to have a superior success rate and similar rates of severe complications compared with ‘PVI-only’ involving 2G-CB. However, we recommend that large, prospective, randomized, controlled studies should be performed in the future to validate our results.

## Supporting information

S1 Table PRISMA checklist. (DOC)
